# Editorial: Reducing the harm of medication–recent trends in pharmacovigilance (volume II)

**DOI:** 10.3389/fphar.2023.1175039

**Published:** 2023-04-05

**Authors:** Elena Ramírez, Miguel González-Muñoz, Chanda Kulkarni, Francisco J. De Abajo

**Affiliations:** ^1^ Clinical Pharmacology Department, La Paz University Hospital-IdiPAZ, Faculty of Medicine, Universidad Autónoma de Madrid, Madrid, Spain; ^2^ Immunology Department, La Paz University Hospital-IdiPAZ, Madrid, Spain; ^3^ iDD Research Solution Pvt.Ltd, Bangalore, India; ^4^ Clinical Pharmacology Unit, University Hospital Príncipe de Asturias, Madrid, Spain; ^5^ Department of Biomedical Sciences (Pharmacology Section), University of Alcalá (IRYCIS), Madrid, Spain

**Keywords:** Adverse drug effect, medication without harm, pharmacovigilance, pharmacoepidemiology, causality assessment, adverse drug reaction

This Research Topic is the *Volume II of Reducing the harm of medication–recent trends in pharmacovigilance*. Ramírez et al. (Ramírez E, González-Munoz M, Kulkarni C, de Abajo FJ. Editorial: Reducing the Harm of Medication-Recent Trends in Pharmacovigilance. Front Pharmacol. 2022 Aug 30;13:964125. doi: 10.3389/fphar.2022.964125). In 2017, the World Health Organization (WHO) launched the third global health challenge, Drugs Without Harm, to achieve a reduction in harm related to iatrogenic drugs by 50% in 5 years. In this Research Topic, our objective was to evaluate strategies to improve the safety in the use of medicines to achieve this objective.

A set of standards, regulations, guidelines and standard operating procedures constitute the fundamental basis of an efficient pharmacovigilance system as part of health policies. Several nations implemented pharmacovigilance systems in the early 1960s and continually introduce new legislation to strengthen their existing medication safety systems. However, the development and implementation of pharmacovigilance systems is very uneven throughout the world. Khan et al. performed a semi-structured exploratory interview with stakeholders to assess their perceptions of the current adverse drug reactions (ADR) reporting system and to identify pharmacovigilance policy issues and effective coordination issues in Pakistan. The results obtained are similar to those of other low- and middle-income countries that identify the lack of a regulatory framework as the main gap in the reporting system for ADRs. These findings highlight differences compared to the pharmacovigilance systems of high-income countries. Rehman et al. performed a survey to determine the perception and effects of intervention on patients regarding ADRs in public hospitals in Islamabad revealed that most of the participants were interested in medical consultation for medication use; some were willing to report ADRs in the future and called for the establishment of a hospital-level pharmacovigilance system. Pakistan felt the need for an effective and robust pharmacovigilance system after one of the deadliest medication-related tragedies that caused more than 300 deaths in 2012. The country established its national pharmacovigilance system center in 2015 and joined the WHO International Drug Program Monitoring in 2018 as a full member. Khan et al. in a descriptive cross-sectional study conducted by providing a questionnaire administered by an interviewer from the pharmacovigilance system through a convenience sampling method using the Indicator-Based Pharmacovigilance Assessment Tool concluded that despite receiving funding from the Global Fund, none of the National Public Health Programs have pharmacovigilance system centers or associated activities. A two-phase strategy is proposed encompassing non-financial and financial interventions to improve pharmacovigilance system systems at the national, provincial, Public Health Programs and hospital levels.

The databases of adverse event reporting systems are public tools available on the web that provide access to search for information related to adverse events in humans. FDA Adverse Event Reporting System (FAERS) is a public web-based tool providing access to search for information related to human adverse events reported to the FDA by the pharmaceutical industry, healthcare providers and consumers. This type of database with all its limitations (duplicate reports where the same report was submitted was submitted by a consumer and by the sponsor, there is no certainty that the reported event was due to the product) offers interested parties stakeholders possibilities for signals mining of possible adverse reactions of many medications. A FAERS-based study was conducted to compare adverse reaction reports and bleeding signals for ticagrelor and clopidogrel. Tang et al., using system organ classes and preferred terms from the Medical Dictionary of Regulatory Activity, analyzed the adverse reaction signals of ticagrelor and clopidogrel.

Most hospitals participate in pharmacovigilance through spontaneous reporting systems. However, spontaneous reporting systems have limitations, such as the difficulty of recognizing ADRs, the uncontrolled nature of the reporting method, and underreporting. For these reasons, retrospective and prospective surveillance methods are considered more effective than spontaneous reporting systems. Valdés-Garicano et al. retrospectively evaluated the performance of a proactive pharmacovigilance system using laboratory alerts as a method to detect serious ADRs using hyponatremia and rhabdomyolysis as case studies. The authors found moderate sensitivity and high specificity for both ADRs.

Diagnosis of delayed-type ADRs is complex and is usually done after recovery. The ADR study includes medical history, causality algorithms, skin tests, and suspected medication rechallenge tests, and helps to identify the immunological mechanisms and culprit medications involved. The identification of the guilty medication is of great importance in the diagnosis, allowing an adequate management of the patient and avoiding a possible re-exposure in the future with serious consequences. The diagnosis based on the clinical history is especially difficult and in many cases it is not easy to establish an accurate time sequence between the administration of the medication and the onset of adverse symptoms. The information that skin tests can provide in the diagnosis of immune-mediated ADRs is limited because their sensitivity and specificity depend on the medication and the clinical manifestations, and their sensitivity is low. Therefore, the gold standard for confirming the diagnosis of severe ADRs and identifying the culprit medication is re-exposure to the suspected medications. However, it raises serious ethical concerns in severe ADRs. One of the approaches that has been explored to improve the diagnosis of ADRs is *in vitro* tests that are safe for patients. Two papers addressed the use of *in vitro* tests in the identification of the culprit medication involved in hypersensitivity ADRs. Bellon et al. carried out a case-control study to evaluate the diagnostic tools in medication induced eosinophilia and systemic symptoms (DRESS) induced by vancomycin in Spanish cases. The evaluation included causality algorithms, the lymphocyte transformation test, and HLA testing. The results confirmed the association of the HLA-A*32:01 risk allele with vancomycin-induced DRESS and support lymphocyte transformation test as a reliable tool for determining vancomycin sensitization. Elzagallaai et al. evaluated the lymphocyte toxicity assay to diagnose and capture a serum sickness-like reaction due to ß-lactam antibiotics. The authors found that there was a significant concentration-dependent increase in cell death in cells isolated from patients compared to cells from healthy controls. The results of both studies suggest that *in vitro* tests could play a role in the diagnosis of hypersensitivity ADRs.

The search for predictors of medication-related problems (MRPs) is an approach that may improve current knowledge regarding the prediction of adverse drug events. Taylor et al. have developed two tools to identify patient, medication, and emergency department (ED) presentation related predictors for MRPs across the continuum of ED care that may require specialist input to identify, manage or prevent. These screening tools were applied (or implemented) at and during the ED presentation (Presentation Tool), and shortly after emergency department or short-stay unit (SSU) discharge (Discharge Tool). Preliminary scoring cutoffs and associated screening tool performance have been proposed. The authors state that MRP predictors are readily available at the bedside and can be used to detect patients at increased risk on presentation to the ED and upon discharge from the ED or SSU at community.

Potentially inappropriate medicines (PIMs) are a major concern in pharmacovigilance and are a well-known public health problem. In this volume, two papers related to potentially inappropriate medication in cancer patients were presented. China is currently the country with the largest population of elderly people with cancer in the world, and cancer, as a chronic disease, places a heavy burden on the elderly. Older cancer patients may suffer from a higher rate of comorbidity, frailty, and geriatric syndrome, putting them at high risk for polypharmacy and PIM use. In the multicenter cross-sectional study, Wang et al. evaluated potentially hazardous drug-drug interactions (DDIs) associated with prescribed oral antineoplastic agents in tertiary care teaching hospital settings without computerized DDI detection programs. Potentially hazardous DDI associated with oral antineoplastic agents were analyzed by using the United States Food and Drug Administration-approved labeling. Nearly 300 DDIs were identified in about 14,000 enrolled patients, with proton pump inhibitors, dexamethasone, and fluoroquinolones being the most frequently dangerous DDIs involved with oral antineoplastic agents. Multivariate analysis revealed younger age, increasing number of medications, and targeted therapy-treated patients were the main risk factors for a DDI. In the other study with cancer patients Tian et al. evaluated the use of potentially inappropriate medication in elderly patients seen in tertiary hospital outpatients with cancer with multimorbidity according to Chinese Geriatrics Association criteria, American Geriatrics Society (AGS)/Beers criteria and the Screening Tool for Prescribing for the Elderly (STOPP) and the Screening Tool to Alert the Right Treatment (START) criteria. The authors found a high prevalence of PIM use in older Chinese cancer outpatients with multimorbidity and low to moderate concordance among the three criteria used. The low concordance between the different criteria highlights the need to develop special PIM detection criteria for older cancer patients.

Evidence-based medicine integrates clinical experience and patient values and aims to use the best evidence to make decisions about the care of individual patients. The patients’ values, which reflect their subjective cognition and demand, have been proposed to be considered as a reliable clinical guide. In the case of pediatric patients, their guardians are responsible for their values. Yang et al. conducted a cross-sectional survey for pediatricians and guardians of children with tic disorders in Myanland, China, Macao, and Hong Kong to analyze information on physician behavior and medication choices and on the Guardians’ knowledge of tic disorder, medical treatment behaviors, and medication. Options and needs. The study revealed that pediatricians in China often follow clinical guidelines when selecting tic disorder medications, but rarely consider guardians’ preferences, highlighting a gap in treatment optimization. In addition, the patients’ guardians lack sufficient knowledge about tic disorders and medication options, requiring more physician-initiated dialogue.

Randomized Controlled Trials (RCTs) are considered the most scientifically rigorous method for regulatory decision making. However, real-world evidence (RWE) is playing an increasing role in healthcare decisions. RWE enables monitoring of post-marketing safety and the assessment of comparative treatment effectiveness, which can be of utmost importance to develop guidelines and decision support tools for use in clinical practice. Jang et al. proposed that RWE has the potential to provide evidence for future regulatory decision-making in an environment where RCTs cannot be performed. Its objective was to investigate to what extent the safety of empagliflozin from the RWE study in Korea is different from that of the RCT emulating the design of a foreign RCT. The results of their study suggest that RWE emulating foreign RCTs has the potential to provide evidence for future regulatory decision-making.

We appreciate the good acceptance of Reducing the Harm of Medication - Recent Trends in Pharmacovigilance series as shown by the interesting contributions to its two volumes. As in the first volume, we make some suggestions to encourage future Pharmacovigilance activities. The first is based on the need for continuous improvement of pharmacovigilance systems, and on the positive involvement of patients in spontaneous reporting systems of possible ADRs. Public adverse event reporting databases (FAERS, Eudravigilance, WHO) make it easier for researchers with data mining skills to use these tools to generate new medication safety signals. As we already said in the first volume, it is necessary to improve diagnostic tools, causality algorithms and other *in vitro* tests in the diagnosis of ADRs. Current methods of diagnosis of severe ADRs, often rare medication hypersensitivity reactions by frequency and mechanism, lack clear diagnostic criteria. Given their safety and good predictive value, lymphocyte transformation test and lymphocyte toxicity assay *in vitro* tests have great potential to be a useful diagnostic tool for severe ADRs. On the other hand, the implementation of tools to reduce potentially dangerous medications in older patients with cancer, drug interactions and inappropriate medications is urgently needed. The electronic medical record has proven to be more useful for evaluating problems already detected, allowing the implementation of prevention and early detection tools that minimize the risk of ADR. The use of large automated databases, including demographic data, diagnoses, procedures, and medication use, can generate real-world evidence (RWE) about the benefits and risks of medications and could even emulate a randomized clinical trial (RCT) with the advantages of providing longer follow-ups of patients with less exclusion criteria and higher external validity. However, RWE-based studies are more prone to systematic errors than RCTs which should be taken into account when assessing causal inference medication. For all these reasons, it is necessary to have a critical mass of specialists for the early detection, diagnosis and management of ADRs who, in collaboration with the authorities and patients, develop and implement the tools that make it possible to reduce severe medication-related harm.

